# Alterations in Phosphorylated CREB Expression in Different Brain Regions following Short- and Long-Term Morphine Exposure: Relationship to Food Intake

**DOI:** 10.1155/2013/764742

**Published:** 2013-08-29

**Authors:** Xiuhai Ren, Kabirullah Lutfy, Michael Mangubat, Monica G. Ferrini, Martin L. Lee, Yanjun Liu, Theodore C. Friedman

**Affiliations:** ^1^Division of Endocrinology, Metabolism and Molecular Medicine, Department of Medicine, Charles R. Drew University of Medicine and Science and UCLA School of Medicine, 1731 E. 120th. Street, Los Angeles, CA 90059, USA; ^2^Department of Pediatrics, Children's Hospital of Los Angeles, University of Southern California, Los Angeles, CA 90027, USA; ^3^College of Pharmacy, Western University of Health Sciences, Pomona, CA 91766, USA

## Abstract

*Background*. Activation of the cyclic adenosine monophosphate (cAMP)/phosphorylated CREB (P-CREB) system in different brain regions has been implicated in mediating opioid tolerance and dependence, while alteration of this system in the lateral hypothalamus (LH) has been suggested to have a role in food intake and body weight. *Methods*. Given that opioids regulate food intake, we measured P-CREB in different brain regions in mice exposed to morphine treatments designed to induce different degrees of tolerance and dependence. *Results*. We found that a single morphine injection or daily morphine injections for 8 days did not influence P-CREB levels, while the escalating dose of morphine regimen raised P-CREB levels only in the ventral tegmental area (VTA). Chronic morphine pellet implantation for 7 days raised P-CREB levels in the LH, VTA, and dorsomedial nucleus of the hypothalamus (DM) but not in the nucleus accumbens and amygdala. Increased P-CREB levels in LH, VTA, and DM following 7-day treatment with morphine pellets and increased P-CREB levels in the VTA following escalating doses of morphine were associated with decreased food intake and body weight. *Conclusion*. The morphine regulation of P-CREB may explain some of the physiological sequelae of opioid exposure including altered food intake and body weight.

## 1. Introduction

Drug addiction is a complex state induced by repeated drug exposure and characterized by compulsive drug-seeking and drug-taking behaviors, despite adverse consequences associated with such behaviors. The processes leading to addiction following drug use and abuse are manifested by adaptations in numerous neuronal circuits in the central nervous system and lead to dependence, tolerance/sensitization, and reinforcement/reward [1]. 

Opioids and other drugs of abuse affect several intracellular messenger systems that are implicated in the addiction process. The most studied of these is CREB, a transcription factor responsive to cAMP that is activated by phosphorylation at serine residue 133 by cAMP-dependent protein kinase A (PKA). Changes in the phosphorylation of CREB result in changes in transcription of genes that are dependent on activation of their CREs that are *cis*-acting enhancer elements on promoters of various genes [2, 3]. CREB is found in most tissues examined, with high levels in the brain [4] and is thought to be an important mediator in substance abuse [5, 6].

In many brain regions, acute administration of *μ*-opioid receptor agonists leads to the inhibition of adenylate cyclase with a resultant decrease in cAMP-dependent protein phosphorylation of CREB [7–9]. In contrast, chronic opioid administration upregulates the cAMP system by increasing adenylyl cyclase activity leading to an increase in the levels of phosphorylated CREB (P-CREB) in some brain regions [10]. Opioid withdrawal further increases CREB phosphorylation [2, 5]. Together, these results (most of which were done in rats) suggest that the actual effects of opioids on phosphorylated CREB may be dependent on the route and duration of opioid administration as well as on which brain region is examined.

The role of P-CREB in food intake, especially as related to opioid exposure, is less well studied. In drosophila, blocking CREB activity in the fat body increased food intake [11]. In mice, fasting induced CREB activation in the lateral hypothalamus (LH) [12] and arcuate nucleus, and the latter correlated with increased neuropeptide Y expression that was blocked by leptin administration [13]. MC4 agonist suppression of food intake is mediated by increased CREB phosphorylation [14]. POMC gene delivery into the hypothalamus of Zucker rats increased phosphorylation of CREB that was accompanied by a reduction in food intake and an attenuation of weight gain [15]. Thus, an inverse relationship between P-CREB in brain regions involved in appetite regulation and food intake exists. Olson and colleagues [16] hypothesized that opioid-induced increases in CREB-mediated transcription in key brain regions may alter drug reward and food intake and showed in rats that viral-mediated expression of CREB-GFP in the LH significantly increased both morphine-conditioned place preference and food intake but did not alter water intake or sucrose preference. 

In the current study, we hypothesized that opioid-induced changes in P-CREB in different brain regions induced by different paradigms of opioid exposure would have reciprocal effects on food intake and body weight, with paradigms leading to increased P-CREB which would decrease food intake and body weight and paradigms leading to reduced P-CREB which would increase food intake and body weight. To test this hypothesis, we measured changes in food intake and body weight as well as changes in P-CREB in various mouse brain regions following different morphine regiments that induce tolerance and/or dependence.

## 2. Materials and Methods

### 2.1. Animals

Male C57BL/6 mice weighing 22–24 g, obtained from Taconic Farms (Germantown, NY), were used for all experiments. Mice were housed 2–4 per cage with each cage receiving the same treatment and having free access to water and food. All experiments using animals were performed in compliance with the NIH Guidelines for the Use of Animals in Research and approved by the Institutional Animal Care and Use Committee (IACUC) at Charles R. Drew University.

### 2.2. Drugs

Morphine sulfate and morphine and placebo pellet were obtained from the National Institute on Drug Abuse (Research Triangle Park, MD, USA). Morphine sulfate was dissolved in normal saline and injected subcutaneously in a volume of 0.1 mL/10 g of mouse body weight, so that the concentration was 10 mg/kg, except as noted.

### 2.3. Opioid Administration

We determined how different opioid regimens regulate the phosphorylation of CREB in various brain regions (see Table 1). In the first condition, we injected mice with saline or morphine once (MOR X1, 10 mg/kg) subcutaneously (s.c.) to examine the effect of single morphine administration on P-CREB. This dose of morphine is expected to induce significant antinociception and reward but not tolerance or dependence [17, 18]. In the second condition, mice received daily single injections of saline or morphine (10 mg/kg, s.c.) for eight consecutive days (MOR X8), in which some degree of tolerance but not dependence was expected [17]. To induce a greater degree of tolerance than single-dose paradigm and also able to observe some degree of dependency, we also designed an escalating dosing paradigm, in which mice received saline or increasing doses of morphine daily (MOR ESC; 10, 20, 20, 40, 40, 80, 160 mg/kg, s.c.). Morphine pellet implantation is commonly used to induce robust tolerance and dependence [19], thus we also used this paradigm, in which mice were implanted with morphine (25 mg) or placebo pellet, to study the effect of a morphine regimen leading to tolerance and dependence on changes in P-CREB levels in different brain regions. We used a 24 h morphine pellet regimen (25 mg implanted s.c.) to mimic high-dose short-term opioid exposure and a seven-day morphine pellet regimen (25 mg pellet implanted s.c. on day 0 and an additional 25 g pellet implanted s.c. on day 4) to mimic longer term high-dose opioid exposure. Morphine and placebo pellets were implanted s.c. at the nape of the neck, while animals were maintained under isoflurane anesthesia (Attane, Minrad INC, Bethlehem, PA, USA). Saline-treated mice from MOR X1 and MOR X8 were pooled together since there was no difference in these groups in all the responses measured. For immunohistochemistry experiments (performed on separate groups of mice; see later), mice were deeply anesthetized and perfused with saline and p-formaldehyde 30 min after the last morphine/saline injection, at 24 h after the 24 h morphine pellet or 3 days following the 2nd long-term morphine/placebo implantation.

### 2.4. Naloxone-Precipitated Opioid Withdrawal

Mice received either escalating dose regimen of morphine or morphine pellet for 24 h or 7 days, or corresponding controls were given naloxone (1 mg/kg, s.c.) to precipitate withdrawal, which was determined by counting the mean number of jumps over the next two h.

### 2.5. Hot Plate Assay

At the end of the treatment period, a separate group of mice from each group were tested for baseline hot plate latency at 0900 and injected with morphine (10 mg/kg). Thirty min later, mice were tested again for hot plate latency. Mice were gently placed into an acrylic box with a metal floor that was preheated to 52.5°C, as described previously [20] using a hotplate analgesia meter (Columbus Instruments, Columbus, OH, USA). Vigorous shaking or licking of either hindpaw was used as the nociceptive response. Percent maximum possible effect (%MPE) was calculated as (test latency − baseline latency)/(cut-off − baseline latency) × 100. A cut-off time of 60 sec was imposed as the maximum possible effect in order to prevent tissue damage. Mice that did not respond by 60 sec were considered as responders to the antinociceptive effects of morphine. 

### 2.6. Food Intake and Weight Change in Morphine- or Placebo-Treated Mice

Animals were weighed daily and the weight change from baseline was calculated. The amount of food consumed per mouse was determined daily by weighing the food pellets and dividing it by number of mice/cage. Food intake and body weight were measured during the different morphine regimens (see Table 1) and not during hotplate or withdrawal testing. 

### 2.7. Effects of Morphine Treatment on P-CREB Levels

Thirty min following their last saline/morphine treatment (at 0900), mice were deeply anesthetized with sodium pentobarbital (100 mg/kg, i.p.) and perfused with phosphate buffer saline (PBS) followed by 4% paraformaldehyde (PFA). Untreated naïve mice were also perfused to determine whether the level of P-CREB was altered by exposing animal to the hot plate and/or acute morphine or saline. The brain of each mouse was isolated and placed in ice cold 4% paraformaldehyde (PFA) overnight at 4°C. Brains were then immersed in 25% sucrose until the tissues sank and were then frozen on dry ice. The brain regions of interest were cut into seven-micrometer-thick coronal cryostat sections, and the sections were mounted onto slides and stored at −80°C until staining.

### 2.8. Immunofluorescence Staining of P-CREB and Total CREB

Prior to further processing, endogenous peroxidase activity was quenched by placing the slides in 0.3% H_2_O_2_ for 10 min, followed by rinsing with PBS. Detection of P-CREB and total CREB was then performed with the use of a biotin-conjugated Tyramide Signal Amplification kit (TSA) (Perkin-Elmer Life Sciences, Boston, MA, USA) according to the manufacturer's instructions. In brief, brain slices were incubated for 45 min in TNB blocking buffer (0.1 M Tris-HCl, pH 7.5; 0.15 M NaCl; 0.5% blocking reagent, provided by TSA kit) at room temperature and then incubated with polyclonal rabbit P-CREB (1 : 1000, Upstate, Lake Placid, NY, USA) or polyclonal rabbit total CREB antibody (1 : 1000; Cell Signaling Technology, Danvers, MA, USA) overnight at 4°C. After rinsing with PBS, the sections were incubated with Biotin-SP-conjugated donkey anti-rabbit IgG (Jackson ImmunoResearch Laboratories) at a 1 : 400 dilution in 0.5% blocking reagent (TSA kit) for 60 min, followed by streptavidin-HRP (SA-HRP, 1 : 100, supplied by TSA kit) for 30 min and finally incubated in fluorophore tyramide amplification reagent (provided in the TSA kit) at a concentration of 1 : 50 for 5−10 min. Slides were rinsed in TNT wash buffer (0.1 M Tris-HCl, pH 7.5, 0.15 M NaCl, 0.05% Tween-20). All incubations were performed at room temperature in a humidified container and protected from light. Finally, slides were mounted using Vectashield mounting medium for fluorescence with DAPI (Vector Laboratories). Immunostained sections were analyzed using fluorescence microscopy (Leica Microsystems, Bannockburn, IL, USA). Images were captured with a SPOT RT (Diagnostic Instruments, Sterling Heights, MI, USA) color digital camera attached to the microscope, and the images captured were saved as Tiff files. The number of P-CREB or total CREB staining positive cells was counted by image analysis using Image Pro 4.01 software (Media Cybernetics, Silver Spring, MD, USA). At least 2–4 images taken at 400X of the brain region of interest were analyzed per tissue section, with at least 4 anatomically matched sections per animal and 3 animals per group. The final results represent the number of CREB or P-CREB stained positive cells per brain region of interest.

### 2.9. Data Analyses

Data are expressed as mean ± SEM. The hot plate latency was analyzed using either a one-way or two-way analysis of variance (ANOVA) as appropriate. The Student-Newman-Keuls post hoc test was used to reveal significant differences between various treatments. The food intake and body weight change data and number of P-CREB immune-stained cells showed a nonnormal distribution and were analyzed by the Kruskal-Wallis nonparametric one-way ANOVA. If a significant difference was found, the pairwise subanalyses of the groups were conducted using the Tukey-Kramer method. Significance level was set at *P* < 0.05.

## 3. Results

### 3.1. Chronic Morphine Treatment Induced Tolerance to the Antinociceptive Effect of Morphine

**Figure 1:**
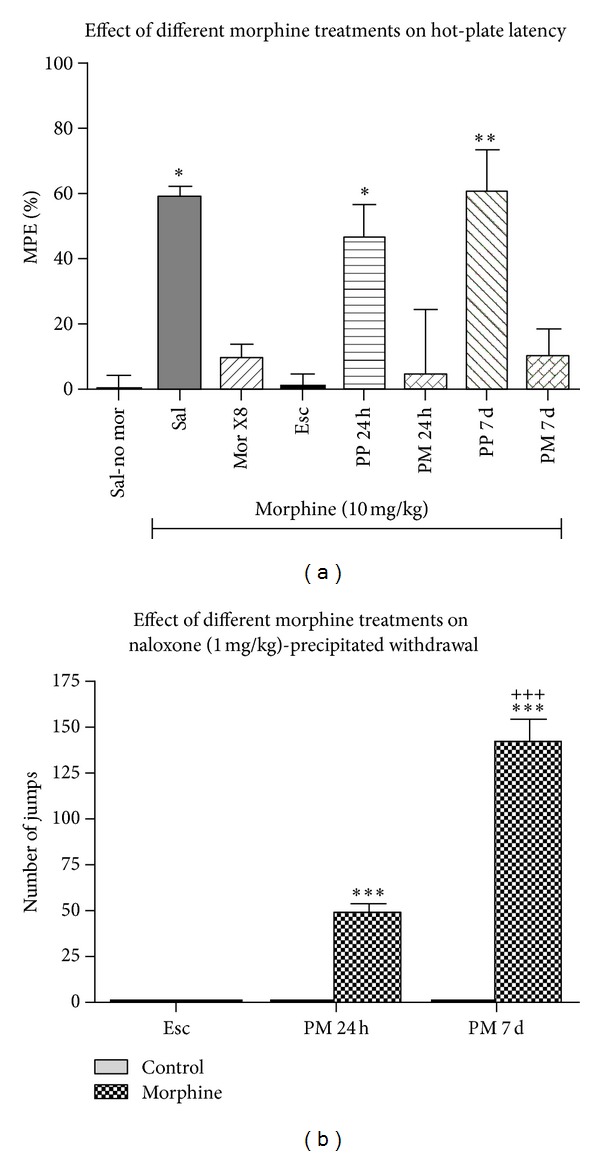
Percent maximum possible effect (%MPE) (a) and naloxone-precipitated withdrawal jumping (b) following different treatments. In hot plate latency testing (a), Sal-no morphine (Sal-no mor) group received saline (s.c.) and was tested for hot plate latency 30 min later. All other groups received their treatment and were then given morphine (10 mg/kg, s.c.) followed by hot plate latency testing 30 min later. See Table 1 for details on the morphine treatment paradigm. ^#^ *P* < 0.05, versus saline-no mor, *P < 0.05, versus saline with morphine given 30 min after. *N* = 5–9 per treatment. In naloxone-precipitated withdrawal (b), mice received either escalating dose of morphine or morphine pellet for 24 h or 7 days or corresponding controls and then received naloxone (1 mg/kg, s.c.) to precipitate withdrawal, which was determined by counting the mean number (±SEM) of jumps over the next two h. The number of jumps in control treated mice was too small to be seen on the figure. ***P < 0.01, versus escalating dose. ^#^ *P* < 0.001, versus PM24h. *N* = 6-7 per treatment.

Figure 1(a) illustrates the ability of morphine to induce antinociception, as measured by changes in hot plate latency and shown here as an increase in %MPE, in mice treated with different morphine regimens and their respective saline/placebo-treated controls. For comparison, %MPE in mice treated with saline but no morphine is also shown (see Table 1 for details of the treatments). One-way ANOVA showed a significant effect of treatment (*F*_7,47_ = 6.36; *P* < 0.0001). Post hoc testing revealed that morphine induced significant antinociception in control (saline-pretreated) mice (Sal group) as well as in mice implanted with placebo pellets (*P* < 0.05 or better, compared to Sal-no Mor, that is, animals treated with saline but no morphine). However, this response was significantly reduced in mice with prior morphine exposure (*P* < 0.05 compared to Mor8X, ESC, PM24 h, or PM7d versus mice pretreated with saline and injected with morphine 30 min prior to the hot plate testing, that is, Sal group) indicating that tolerance developed to the antinociceptive effect of morphine.

### 3.2. Chronic Morphine Treatment Led to Naloxone-Precipitated Withdrawal

Figure 1(b) illustrates number of jumps (a somatic sign of opioid withdrawal) in mice receiving escalating dose morphine or treated with morphine pellet for 24 h or 7 days or their corresponding controls. Two-way ANOVA revealed a significant interaction between treatment (vehicle versus morphine) and group (escalating dose, versus 24 h morphine pellet versus 72 h morphine pellet) (*F*_2,32_ = 120; *P* < 0.0001). Post hoc analysis showed that naloxone induced a significantly higher number of jumps in mice implanted with morphine for 24 or 7 days compared to the mice treated with escalating doses of morphine. Also, we found that the number of jump was significantly greater in mice with longer morphine exposure (*P* < 0.001, compared to 7 d versus 24 h pellet group). There were no jumps in mice receiving vehicle (and not morphine), escalating dose, or placebo pellets and in mice receiving a single dose or 8 daily doses of morphine (data not shown). These results confirm that morphine pellet implantation leads to opioid dependence.

### 3.3. Chronic Morphine Treatment Regulated Body Weight and Food Intake

**Figure 2:**
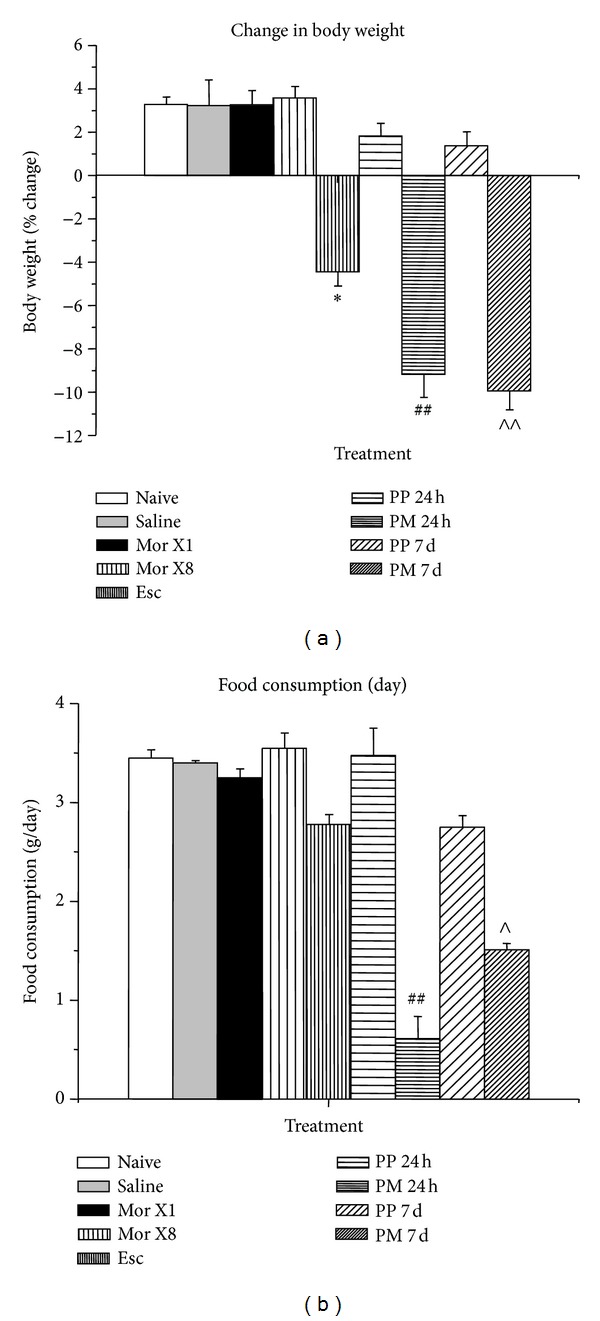
Body weight change (% of initial body weight) (a) and food intake (g/day) (b) following different morphine treatments. See Table 1 for details on the morphine treatment paradigm. *N* = 3-4 per treatment. *P < 0.05, versus saline injection, ^##^ *P* < 0.0001 versus 24 h placebo pellet, ^∧^ *P* < 0.05 versus 7 d placebo pellet, ^∧∧^ *P* < 0.0001 versus 7 d placebo pellet.

One-way nonparametric ANOVA showed a significant effect of treatment on body weight (*χ* ^2^ = 42.6, *df* = 8, *P* = 0.000001) and food consumption (*χ* ^2^ = 32.5, *df* = 8, *P* = 0.00008). Naïve mice not undergoing hot plate testing, saline-treated mice undergoing hot plate testing, and mice treated with a single dose of morphine undergoing hot plate testing all showed similar body weight (Figure 2(a)) and food intake (Figure 2(b)) over a 24 h period. Morphine injection given daily for 8 days was without a significant effect on body weight (Figure 2(a)) and food intake (Figure 2(b)). The escalating dose of morphine for 8 days significantly decreased body weight (*P* < 0.0001) (Figure 2(a)) but not food intake (Figure 2(b)). Both short-term morphine implantation for 24 h and longer term morphine exposure for 7 days significantly decreased body weight (*P* < 0.0001 for both) (Figure 2(a)) and food intake (*P* < 0.0001 for the 24 h pellet and *P* < 0.05 for 7-day pellet) (Figure 2(b)) compared to implanted mice that were not were not different in body weight and food intake compared to naïve controls.

### 3.4. Short-Term and Long-Term Morphine Treatments Differentially Altered P-CREB Levels

**Figure 3:**
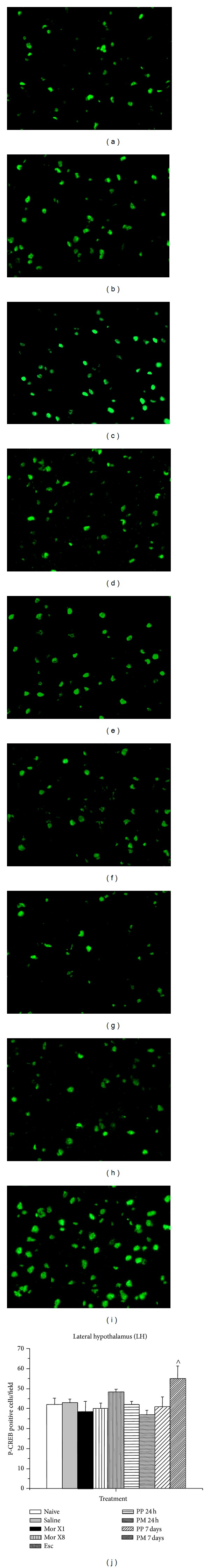
Changes in P-CREB staining in the LH following different morphine treatments ((a) group 1, naïve; (b) group 2, saline; (c) group 3, MorX1; (d) group 5, MorX8; (e) group 6, Esc; (f) group 7, PP24 h; (g) group 8, PM24 h; (h) group 9, PP7d; (i) group 10 PM7d) as determined by immunofluorescence (200X). Quantification of P-CREB (j). See Table 1 for details on the morphine treatment paradigm. *N* = 6–9 per treatment.  ^∧^ *P* < 0.05 versus 7 d placebo pellet.

**Figure 4:**
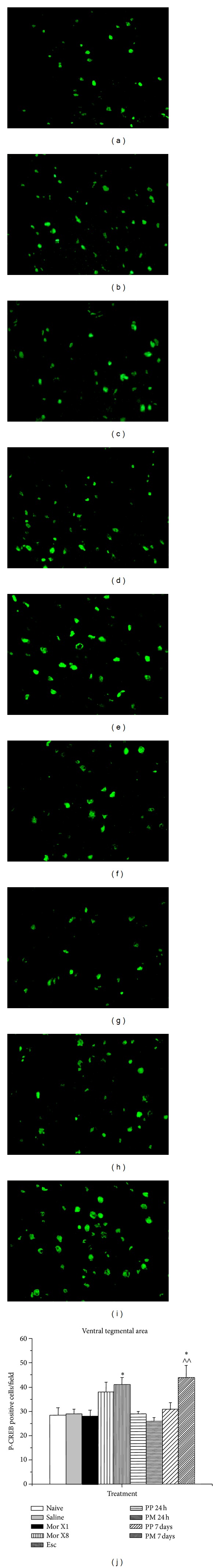
Changes in P-CREB staining in the VTA following different morphine treatments ((a) group 1, naïve; (b) group 2, saline; (c) group 3, MorX1; (d) group 5, MorX8; (e) group 6, Esc; (f) group 7, PP24 h; (g) group 8, PM24 h; (h) group 9, PP7d; (i) group 10 PM7d) as determined by immunofluorescence (200X). Quantification of P-CREB (j). See Table 1 for details on the morphine treatment paradigm. *N* = 6–9 per treatment. *P < 0.005 versus saline injection, **P < 0.0001 versus saline injection,  ^∧^ *P* < 0.05 versus 7 d placebo pellet.

**Figure 5:**
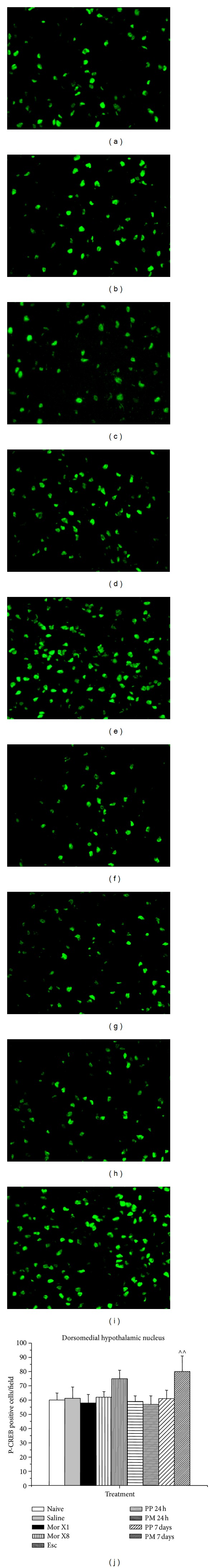
Changes in P-CREB staining in the DM following different morphine treatments ((a) group 1, naïve; (b) group 2, saline; (c) group 3, MorX1; (d) group 5, MorX8; (e) group 6, Esc; (f) group 7, PP24 h; (g) group 8, PM24 h; (h) group 9, PP7d; (i) group 10 PM7d) as determined by immunofluorescence (200X). Quantification of P-CREB (j). See Table 1 for details on the morphine treatment paradigm. **P < 0.01 versus saline injection,  ^∧∧^ *P* < 0.01 versus saline and 7 d placebo pellet.

The levels of P-CREB and CREB in various brain regions were measured following different morphine treatment protocols (Table 1). In the brain regions analyzed, the number of total CREB -positive cells or DAPI-stained cells/field was not affected by any treatment. The p-CREB positive staining was mainly localized in the nucleus of the cells. Naïve mice, mice receiving saline injections for 1 dose, and mice receiving saline injections daily for 8 days all had similar levels of P-CREB immunoreactivity in the nucleus (Figures 3–5, Table 2).

In the LH (Figure 3), one-way nonparametric ANOVA revealed a significant effect of treatment on P-CREB immunostaining (*χ* ^2^ = 23.6, *df* = 8, *P* = 0.003). Daily morphine injections or escalating dose (Esc) for 8 days did not alter P-CREB expression, while long-term morphine implantation for seven days (PM7d) significantly increased P-CREB immunoreactivity compared to placebo treatments (*P* < 0.01). Other treatments did not have a significant effect on the level of P-CREB.

In the ventral tegmental area (VTA) (Figure 4), one-way nonparametric ANOVA revealed a significant effect of treatment on P-CREB immunostaining (*χ* ^2^ = 39.4, *df* = 8, *P* = 0.000004). The escalating dose of morphine (*P* < 0.005) and morphine implantation for seven days (*P* < 0.0001) significantly increased P-CREB immunoreactivity compared to placebo treatments. Short-term treatment did not have a significant effect on the level of P-CREB. 

In the dorsomedial hypothalamic nucleus (DM) (Figure 5), as in VTA, one-way nonparametric ANOVA revealed a significant effect of treatment on P-CREB staining (*χ* ^2^ = 22.3, *df* = 8, *P* = 0.004). Morphine implantation for seven days significantly increased P-CREB immunoreactivity compared to placebo treatments (*P* < 0.01). Other treatments did not have a significant effect on the level of P-CREB.

Table 2 summarizes the effect of different morphine treatment regimens on the three regions described previously (LH, DM, and VTA) as well as the amygdala (AMG) and nucleus accumbens (NAC, also known as ventral striatum). Although the AMG and NAC are important regions in drug addiction, morphine paradigms did not significantly affect P-CREB expression in the AMG (*χ* ^2^ = 8.2, *f* = 8, *P* = 0.42) and NAcc (*χ* ^2^ = 6.88, *df* = 8, *P* = 0.45).

## 4. Discussion

In the current paper, we used different morphine treatment paradigms that led to morphine tolerance without dependence, tolerance and partial dependence, and tolerance and full dependence and were associated with differential regulation of P-CREB expression in various brain regions. In agreement with the literature that chronic morphine increases P-CREB expression in certain brain regions [10], we found that the chronic morphine treatments that led to tolerance and dependence were associated with increased P-CREB expression in the LH, DMH, and VTA, with accompanying decreases in body weight and food intake. In contrast to the literature that acute morphine decreases P-CREB expression in certain brain regions [7–9], we found that a single dose of morphine and exposure to short-term morphine (24 h morphine implantation), while decreasing both food intake and body weight, failed to alter P-CREB expression in none of the brain regions examined. Thus, our findings are only somewhat in agreement with the literature that changes in P-CREB expression and food intake/subsequent body weight are inversely correlated, suggesting that mechanisms other than those involving P-CREB are likely to mediate the effect of morphine on decreasing body weight and food intake. 

The effects of opioids on food intake are controversial. In rodents, both hyperphagia [21–23] and anorexia [24–30] have been reported depending on the dose and treatment regimen of morphine (reviewed in [31]). Leshem [27, 32] showed that morphine has a triphasic effect on feeding. Following injection of 15 mg/kg, morphine suppressed intake during the first h, enhanced intake during the next 3 h, and then suppressed intake again for up to 24 h. In humans, opioid agonists such as methadone and butorphanol tartrate stimulated food intake, while naloxone, an opioid antagonist, acutely reduced food intake in obese or lean humans. Long-term studies with naltrexone, an antagonist similar to naloxone, showed no effect on food intake or body weight [33]. These studies were all performed prior to the discovery of many of the appetite-regulating pathways in the last 15 years [34].

The neuronal substrates governing the effects of opioids on the CREB system and subsequently on behaviors such as food intake are not known. Alterations in P-CREB signaling could alter the sensitivity of neurons to dopamine or norepinephrine and thereby affecting reward [16, 35]. Recent studies have established a role for orexin-expressing neurons in the LH in opioid reward [36] and in modulating stress-induced regulation of opioid reward [37]. This raises the possibility that orexin neurons may mediate the effects of opioid-induced changes in P-CREB on food intake. Alternatively, upregulation of P-CREB specifically in melanocyte-concentrating hormone (MCH) neurons may play a role in opioid-induced regulation of P-CREB and its effects on feeding behavior. We anticipate that future studies employing double immunofluorescence staining for some of these neural substrates and P-CREB and/or mice harboring null mutation of these neuropeptides may provide new insights on the involvement of CREB in morphine-mediated decrease in food intake and weight loss.

 Other candidate genes affected by opioid use that could mediate behaviors such as food intake are the CRE-dependent processing enzymes (prohormone convertases 1/3 and 2) [38, 39] that control the ratio of prohormone to active hormone. We found that short-term morphine exposure downregulated and long-term morphine exposure upregulated rat hypothalamic P-CREB, PC1/3, and PC2 protein levels, and immunofluorescence studies confirmed these regulatory actions of morphine in the paraventricular and dorsomedial nucleus of the hypothalamus [40]. In this earlier paper, we concluded that the downregulation of PC1/3, PC2, and P-CREB by short-term morphine treatment and upregulation by long-term morphine treatment may be a signal mediating the switch from drug use to drug abuse. The pattern of regulation of hypothalamic P-CREB by short- and long-term morphine was somewhat different from what we found in the current paper, in that short-term opioid treatment had no effect on P-CREB in the pituitary, while they decreased P-CREB in the hypothalamus. In contradistinction, long-term opioid treatment increased P-CREB both in the pituitary and the hypothalamus.

Using microarray techniques, our laboratory showed that long-term morphine treatment increased hypothalamic and pituitary NPY and AgRP expression as well as the pituitary CART expression, whereas the same treatment decreased pituitary NPY receptor and hypothalamic peptide YY expression [41]. In contrast, short-term morphine exposure decreased the pituitary leptin receptor and hypothalamic and pituitary adiponutrin expression. Overall, we found a strong correlation between morphine-induced alterations in food intake and regulations of genes involved in this process.

The strengths of the current paper include using different paradigms of morphine treatment that gave different degrees of tolerance and dependence and that the morphine treatments leading to the more pronounced opioid tolerance and dependence were associated with an increase in P-CREB expression and decreased food intake. The finding that P-CREB regulation did not occur in all regions suggests some specificity of the effect of morphine on regulating P-CREB and food intake. A caveat of the study is that the association between opioid-induced changes in P-CREB and food intake were correlative and may not be causative. Additionally, we are unable to precisely determine which brain region is mainly responsible for the change in food intake, although we expect it to be the LH. Finally, we only analyzed the number of P-CREB-positive cells and not the intensity of the immunostaining because of the low reliability of determining the integrated optical density by immunofluorescence. To overcome these shortcomings, future studies could inject dominant negative CREB vectors into different brain regions to see if they block the effect of morphine on food intake. Of note, Olson and colleagues [16] found that injection of a CREB vector into the LH increased food intake, while an injection of a dominant negative CREB vector did not alter food intake. They did not examine the effects of CREB manipulation on food intake following opioids.

## 5. Conclusion 

In conclusion, we found that different morphine regimens regulated P-CREB expression in a manner consistent with their effect on addiction parameters, such as morphine tolerance and dependence. The changes in P-CREB following morphine in brain regions implicated in food intake such as the LH were inversely correlated with the changes in food intake and body weight. Further studies are needed to understand the physiological consequences of the opioid-induced regulation of P-CREB and the mechanisms on how this regulation is related to food intake and reward. 

## Figures and Tables

**Table 1:** Morphine treatment schedule.

Group 1: Naïve (without any treatment)	
Group 2: Saline single injection or 1 injection each day for 8 days, s.c. (data combined as there was no difference between the two groups)	
Group 3: MorX1 (morphine10 mg/kg, one injection, s.c.)	
Group 4: Sal X8 (saline, 8 injections, 1 injection each day, for 8 days, s.c.)	
Group 5: MorX8 (morphine10 mg/kg, 1 injection each day × 8 days, s.c.)	
Group 6: Esc, morphine escalating dose (10, 20, 20, 40, 40, 80, 160 mg/kg, s.c.)	
Group 7: PP24h, acute implantation of placebo pellet, sacrificed 24 h later	
Group 8: PM24h, acute implantation of morphine pellet (25 mg), sacrificed 24 h later	
Group 9: PP7d, chronic implantation of placebo pellets (on day 1 and day 4) and sacrificed on day 7	
Group 10: PM7d, chronic implantation of morphine pellets (25 mg) (on day 1 and day 4) and sacrificed on day 7	

**Table 2:** Summary of changes in CREB phosphorylation in different brain regions following morphine treatment.

Brain region	Naïve	Saline	Morx1	Morx8	Esc	Acute PP	Acute MP	Chronic PP	Chronic MP
AMG	C	—	—	—	—	C	—	C	—
DM	C	—	—	—	—	C	—	C	↑
LH	C	—	—	—	—	C	—	C	↑
NAcc	C	—	—	—	—	C	—	C	—
VTA	C	—	—	—	↑	C	—	C	↑

C: control; —: no change from appropriate control (saline versus naïve, Morx1, Morx8 and Esc versus saline, acute MP versus acute PP, chronic MP versus chronic MP), Morx1 single injection of morphine, Morx8, daily injection of morphine for 8 days, acute PP, 24 h placebo pellet, acute MP, 24 h morphine pellet, chronic PP, 7 day placebo pellet, chronic MP, and 7 day morphine pellet. AMG: amygdala; DM: dorsomedial hypothalamic nucleus; LH: lateral hypothalamus; NAcc: nucleus Accumbens; VTA: ventral tegmental area.
